# Editorial: Artificial intelligence for extracting phenotypic features and disease subtyping applied to single-cell sequencing data

**DOI:** 10.3389/fgene.2022.1083719

**Published:** 2023-01-04

**Authors:** Saurav Mallik, Anirban Mukhopadhyay, Aimin Li, Gabriel J Odom, Namrata Tomar

**Affiliations:** ^1^ Department of Environmental Health, Harvard T. H. Chan School of Public Health, Boston, MA, United States; ^2^ Department of Computer Science and Engineering, University of Kalyani, Kalyani, West Bengal, India; ^3^ School of Computer Science and Engineering, Xi’an University of Technology, Xi’an, China; ^4^ Department of Biostatistics, Florida International University’s Stempel College of Public Health, Miami, FL, United States; ^5^ Department of Biomedical Engineering, Medical College of Wisconsin, Milwaukee, WI, United States

**Keywords:** artificial intelligence, feature selection, single cell sequencing data, classifier, clustering, dimensionality reduction, biomarker discovery

With the advent of single-cell sequencing mechanisms, different kinds of omics data (*viz.*, epigenomic, genomic or transcriptomic data profiles) consisting of a lot of individual cells in the parallel-basis, have been generated. Major challenge of the single-cell mechanisms is complexity of genomic or epigenomic information that creates the problem of heterogeneity generating big obstacles to obtain best possible outcomes (or, prediction) in various diseases, mainly for precision oncology. Thus, new artificial intelligence (AI) methods are increasingly proposed and applied to exploit the useful information gathered in those data yielding better accuracy, flexibility, user-friendliness as well as scalability, and finally deliver the effective medicine of precision useful to the patients of the disease. However, the major goal of this Research Topic is to provide the opportunity to the computational biologists and clinical researchers from around the world to contribute their innovative idea and experience towards various underlying problems and their potential solutions in the sphere of computational research in next-generation sequencing data especially, single cell sequencing data for numerous complex diseases (Mallik and Zhao).

Our Research Topic basically covers several new AI tools and frameworks applied to single-cell sequencing data where various computational problems regarding transcriptomics (*viz.*, gene-gene interactions, cell type detection, cell-cell interactions, biomarker discovery, disease classification, disease subtyping and clinical diagnosis) are explored with the advanced translational twist. More emphases are provided to sort out complex real-life medically relevant problems and disorders. Altogether, these manuscripts present kinds of cutting-edge subjects in single-cell sequencing, suggesting that single-cell sequencing data analysis has an increasingly significant role in advancing disease medical application of computational biology. Additionally, it has been observed that new artificial intelligence algorithms are applied to single-cell sequencing data to resolve multiple complex problems that tend to optimize them.

Ligand-Receptor (LR) Hunting methodology employing Random Forest classifier model was developed to discover cell-cell interactions based on single-cell sequencing (scRNA-seq) gene expression data (Lu et al.). LR Hunting discovered the validated interactions between the myeloid cells and CD4^+^ T-cell in Triple Negative Breast Cancer (TNBC). Also, a convolutional neural network (CNN)-based framework for subtype identification was developed where both the transcriptome and methylome profiles were applied to construct the internal classification framework, that clearly separated three subtypes of Glioblastoma (GBM) with higher classification accuracy (Munquad et al.). Another work concerning subtype-specific predictive biomarker discovery was conducted that is applicable to the disease diagnosis and treatment (Munquad et al.). However, it is well-known that identifying potential biomarkers in every cell cluster is inconvenient and unfamiliar that obstructs the systematic study and analysis of single-cell sequencing data. Therefore, to resolve the underlying challenge, a regularized multi-task learning (RMTL) based framework was developed for the simultaneously prediction of the subpopulation related to a specified cell type (Upadhyay and Ray). The regularization strategy was utilized to modulate the multi-task model, smoothing the loss function and thus minimizing the time complexity of the model. Interestingly, artificial neural network (ANN)-based deep learning model was proposed to classify breast cancer samples where the performance of various well-known classifiers or related strategies were evaluated and compared accordingly (Jia et al.). Furthermore, another work concerning cluster-specific frequent biomarker identification through the consecutive utilization of two strategies (*viz.*, dimensionality reduction and Louvain hierarchical agglomerative clustering) was performed from scRNA-seq data (Seth et al.).

Differential network analysis can be able to learn how gene-gene interactions will change along with different biological conditions. A sparse hierarchical Bayesian factor model was developed for scRNA-seq data from different biological conditions, while the methodology utilized a latent factor structure to make impact on the gene expression for cells for helping account for the zero-inflation as well as high cell-to-cell variability (Sekula et al.). Another computational framework was to investigate the molecular interaction techniques in melanoma where a melanoma-specific cell–cell interaction network was built, and a consensus clustering based on network embedding had been applied (Wang et al.).

Finally, it is noticed that majority of the published works in this Research Topic basically cover the drop-out finding, cell-cell interactions, cancer classification model, gene regulatory network inference, cluster-specific frequent biomarker discovery and multimodal data integration. The developed software, tools or methods facilitate the interpretation of the multi-omics data profiles that can improve both the industrial and academic sectors. Moreover, it is highly expected that the upcoming computational tools will be benevolent for biomedical and clinical researchers to understand the applications of AI and machine learning to enhance the quality of research in complex disease detection as well as solve other critical emergency clinical issues (Mallik et al. **)**.

